# Melanoma antigen genes A1 and A3 as predictors of treatment response and survival in HCV-associated hepatocellular carcinoma: a prospective study

**DOI:** 10.1186/s12876-025-04574-8

**Published:** 2026-01-22

**Authors:** Hoda Elgamal, Dina Elhammady, Salwa M. Abo El-Khair, Adel El-Badrawy, Amr Samir, Khaled Farid, Hatem El Alfy

**Affiliations:** 1 Tropical Medicine Department, Faculty of Medicine, Mansoura University, Mansoura, Egypt; 2 Medical Biochemistry and Molecular Biology Department, Faculty of Medicine, Mansoura University, Mansoura, Egypt; 3 Diagnostic and Interventional Radiology Department, Faculty of Medicine, Mansoura University, Mansoura, Egypt

**Keywords:** HCC, MAGE genes

## Abstract

**Introduction:**

Hepatocellular carcinoma (HCC) accounts for approximately 90% of all primary liver cancers resulting in approximately 830,000 deaths in 2020, making HCC the sixth most common cancer globally. Several members of the Melanoma Antigen Gene (MAGE) family, such as MAGE-A1 and A3 genes of the MAGE I-A subfamily, are abnormally expressed in a variety of cancers including melanomas, colorectal cancer, non-small cell lung cancer, gliomas, HCC, prostate, and breast cancers. In addition, they have been linked to tumor stemness and, therefore, may predict therapeutic response and prognosis.

**Aim of the study:**

To investigate the expression pattern of MAGE-A1 and A3 genes in HCC patients and to evaluate their prognostic value.

**Subjects and methods:**

This prospective four-year case-control study was conducted from 2020 to 2024 on 95 subjects classified into three groups. Group (A): Fifty patients with HCC developed on top of hepatitis C virus (HCV) - induced liver cirrhosis, Group (B): 30 patients with post-HCV liver cirrhosis and Group (C): 15 healthy control subjects. Quantitative real-time polymerase chain reaction (qRT-PCR) was used to evaluate the expression of MAGE-A1 and A3 genes in peripheral blood sample after peripheral blood mononuclear cells (PBMCs) isolation. Patients were followed up over 2 years duration for assessment of treatment response.

**Results:**

At cutoff levels 11.54 and 11.39 MAGE-A1 and MAGE-A3, respectively, could distinguish HCC from liver cirrhosis with area under the curve (AUC) of 0.781 and 0.966, respectively (*P* ≤ 0.001). Regarding treatment response, MAGE-A1 and A3 showed significantly higher expression in patients with progressive disease (16.16 and 17.79, respectively) compared to complete response patients (9.21 and 13.25, respectively). Finally, when assessing overall survival (OS) in HCC patients multivariate analysis showed that independent factors for shorter OS were increased MAGE-A1 (HR: 9.407, P: 0.048), high MAGE-A3 (HR: 9.199, P: 0.042) and elevation of both alpha fetoprotein (AFP) and MAGE-A3 (HR: 10.681, P: 0.039).

**Conclusion:**

MAGE-A1 and A3 genes can be used as diagnostic and prognostic markers in HCC patients predicting therapeutic response and overall survival.

**Supplementary Information:**

The online version contains supplementary material available at 10.1186/s12876-025-04574-8.

## Introduction

 Hepatocellular carcinoma (HCC) accounts for approximately 90% of primary liver cancers [1] resulting in about 830,000 deaths by 2020 [2], making it the third leading cause of cancer-related death, despite being the sixth most common cancer worldwide [3].

Depending on worldwide geography, incidence of HCC varies greatly according to prevalence of predisposing risk factors to HCC development [4]. Newly diagnosed cases of HCC in developing countries are primarily attributed to pre-existing liver cirrhosis or hepatitis B virus (HBV) chronic hepatitis [5], with hepatitis C virus (HCV) being the main contributory factor for development of HCC in Egypt [6].

Melanoma Antigen Gene (MAGE) protein was first identified in a patient with melanoma due to its antigenic characteristics [7]. Several members of the MAGE family are aberrantly expressed in a variety of cancers including melanomas, colorectal cancer, non-small cell lung cancer, gliomas, HCC, prostate, and breast cancers [8, 9].

Based on location and pattern of gene expression, MAGE family members are subdivided into MAGE I and MAGE II groups [10]. The MAGE I family group includes MAGE A, B and C subfamily members located on the X chromosome and are expressed primarily in testicular tissue in addition to their aberrant expression in different tumors, while the MAGE II subfamily members include MAGE D, E, F, G, H, L and Necdin genes not positioned on the X chromosome and are expressed in a variety of different tissues [11]. MAGE-A1 and MAGE-A3, either individually or in tandem, are two of the most common MAGE gene members frequently expressed in different neoplasms, as well as in nonmalignant cells such as the placenta and testes, a similarity they share with other MAGE members [12].

In addition to the role of different MAGE genes in tumor stemness and cancer stem cell (CSC) detection, they can also be used in assessing patient prognosis by predicting therapeutic response in different cancers [13].

Although the role of MAGE gene family in different malignancies is not innovative; this work may add further value over previous studies through linking expression levels of MAGE genes to treatment response in HCC patients, in addition to its role in predicting overall survival (OS). Therefore, the aim of this study was to investigate the expression pattern of MAGE-A1 and MAGE-A3 genes in patients with HCC and to assess its prognostic value.

## Materials and methods

### Ethical consideration

After obtaining the approval from the Institutional Research Board (IRB) Committee of the Mansoura Faculty of Medicine (MD.20.11.381), a written informed consent was obtained from all included liver cirrhosis and HCC patients.

### Study subjects

**Fig. 1:**
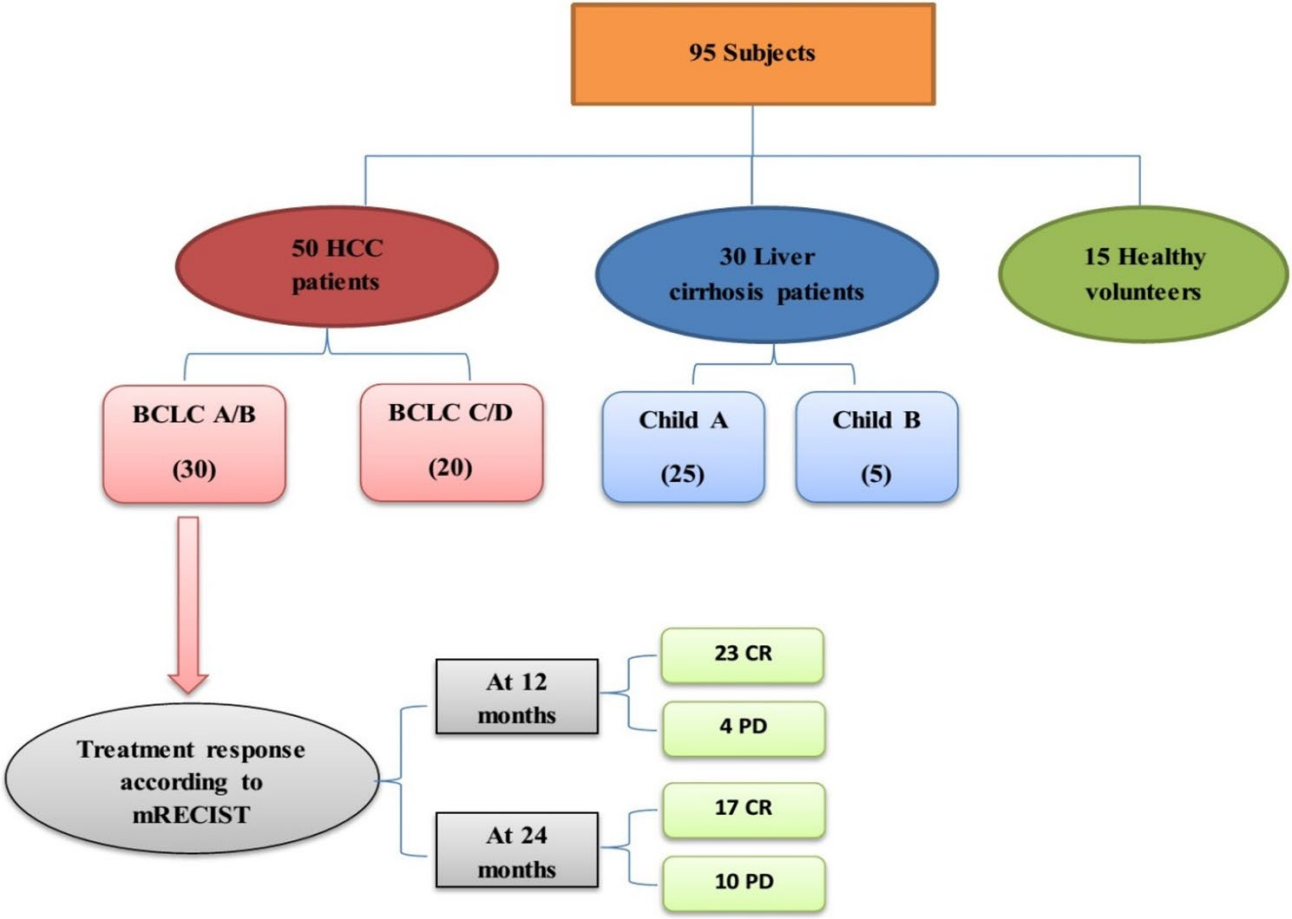
Flowchart of included HCC, cirrhotic patients and healthy subjects. BCLC: Barcelona-Clinic Liver Cancer, CR: complete response, HCC: hepatocellular carcinoma, mRECIST: modified Response Evaluation Criteria in Solid Tumors, PD: progressive disease

This prospective four-year case-control study was conducted from 2020 to 2024 on 95 subjects classified into three groups. *Group A* comprised fifty patients with HCC developed on top of hepatitis C virus (HCV) - induced liver cirrhosis, (subclassified into 30 BCLC A/B & 20 BCLC C/D), while *Group B* consisted of 30 patients with post-HCV liver cirrhosis (subclassified into 25 Child A & 5 Child B) and *Group C* included 15 healthy individuals considered the control subjects for assessment of gene expression levels (Fig. 1).

HCC diagnosis and tumor staging were based on tri-phasic pelvi-abdominal imaging (computerized topography or magnetic resonance imaging), while treatment modality allocation was decided based on Barcelona-Clinic Liver Cancer (BCLC) 2022 update [14], cirrhotic patients were classified according to child-Pugh classification [15].

Based on the European Association for the Study of the LIVER (EASL) clinical practice guidelines for management of hepatocellular carcinoma [16], all studied patients were followed up for 2 subsequent years and response to therapy was finally assessed based on modified Response Evaluation Criteria in Solid Tumors (mRECIST) [17].

Sample size was calculated using online sample size calculator (https://riskcalc.org/samplesize/) with anticipated prevalence of MAGE-A1 and MAGE-A3 gene expression in HCC as regard cirrhosis and healthy volunteers and level of alpha error of 5% and study power of 90%. A minimal sample size required for the study is calculated to be 36 for HCC group, 9 for cirrhotic group and 9 for control group. For possible dropout sample size was increased to 50 patients with HCC, 30 patients with Cirrhosis and 15 healthy control subjects.

### Specimen collection

A 5-mL venous blood sample was collected from all participants and added to EDTA containing vacutainers. Samples were stored in −80 °C freezer until full samples collection and hence processed.

### Molecular study of gene expression of MAGE-A1 and MAGE-A3 genes by quantitative real-time PCR

Blood samples were used for the assessment of gene expression of the target genes (MAGE-A1 and MAGE-A3), in addition to Glyceraldehyde-3-phosphate dehydrogenase (GAPDH) as a control gene, by quantitative real-time PCR (qRT-PCR). Peripheral blood mononuclear cells (PBMCs) were isolated from whole blood using Biocoll separating solution purchased from Biochrome, England (Cat No. L 6113) according to the method of Noble and Cutts, 1967 [18]. Total ribonucleic acid (RNA) isolation from PBMCs was done by the method of Chomczynski, 1993 [19] using a QIAzol™ reagent kit (Catalog no. 79306, Qiagen, Germany). The NanoDropTM 2000 Spectrophotometer was utilized to determine the amount and purity of extracted RNA (Thermo Scientific, USA) as per the manufacturer’s guidelines. A260/A280 ratio represents the purity of the extracted Deoxyribonucleic acid (DNA) (~1.8 is generally accepted). Agarose gel electrophoresis was done to assess the integrity of RNA and to detect any degree of DNA contamination.

Reverse transcription reaction was used to synthesize complementary DNA (cDNA) with ≈250ng total RNA using Maxima First Strand cDNA Synthesis Kit (Thermo Scientiଁc, Waltham, MA, USA, cat No. #K1641). The mRNA expressions were quantiଁed by RT-PCR using the Applied Biosystem 7500, RT-PCR detection system (Life Technology, Carlsbad, CA, USA), and Applied Biosystem SYBR^®^ Green PCR Master Mix (2X) (Life Technology, USA, cat. No. 4344463). The RT-PCR reaction involved enzyme activation at 95 °C for 10 min, then 40 cycles of two-step cycling, including template denaturation at 95 °C (15 s), then, annealing/extension at 60 °C (1 min).

The primer sequences used were as follows: MAGE-A1: Forward (5`- ACA GAG GAG CAC CAA GGA GAA G −3`)/Reverse (5`- AGT TGA TGG TAG TGG GAA AGG C −3**`)**; MAGE-A3: Forward (5`- CGG AGG AGC ACT GAA GGA GAA G 3`)/Reverse (5`- CCT CCT CTT CTT CGT TGC TGG −3`); GAPDH: Forward (5`- GTC AAG GCT GAG AAC GGG AA −3`)/Reverse (5`- AAA TGA GCC CCA GCC TTC TC −3`) [20].

The specificity of the primer sequences was checked using the Primer-BLAST program (https://www.ncbi.nlm.nih.gov/tools/primer-blast). Melting curve analysis was performed to check the specificity of the amplified PCR products. The detection limit and assay sensitivity were determined by the software of using real-time PCR technology (Applied Biosystem 7500, RT-PCR detection system, Life Technology, Carlsbad, CA, USA). (The Ct-threshold of the real-time PCR reactions was set at 0.158 for MAGE-A1 gene, at 0.179 for MAGE-A3 and at 0.146 for GAPDH gene. GAPDH was used as an internal control to normalize the expression of the analyzed genes. Calculations were done according to the comparative 2-ΔΔct method [21]. The data were presented as Relative Quantity (RQ) of target mRNA, normalized in respect to the housekeeping gene (GAPDH gene) mRNA and relative to the control sample.

### Statistical analysis

Data were entered and analyzed using IBM-SPSS software (IBM Corp. Released 2017. IBM SPSS Statistics for Windows, Version 25.0. Armonk, NY: IBM Corp.).

Bonferroni correction was applied to control for multiple testing; however, because several comparisons yielded very small p-values (*p* < 0.001), the corrected results remained significant.

Stepwise Cox regression confirmed MAGE-A1, MAGE-A3, and AFP as the most stable independent predictors of shorter OS.

## Results

**Table 1:** Demographic data among the studied groups and type of therapy in HCC patients

			Control *N*=15	Cirrhotic *N*=30	HCC *N*=50	*P*
Age (years)*		**Mean ± SD**	**59.4 ± 6.20**	**61.5 ± 6.51**	**63.4 ± 7.17**	0.124
**Gender**	**Male**	**N/(%)**	**9 (60.0%)**	**16 (53.3%)**	**33 (66.0%)**	0.529
	**Female**	**N/(%)**	**6 (40.0%)**	**14 (46.7%)**	**17 (34.0%)**	
**DM**	**Present**	**N/(%)**	**----**	**11 (36.7%)**	**9 (18.0%)**	0.062
**HTN**	**Present**	**N/(%)**	**----**	**8 (26.7%)**	**7 (14.0%)**	0.160
**Type of therapy**:						
**PEI**		**(N (%))**	**----**	**----**	**1 (3.3%)**	**----**
**TACE**		**(N (%))**	**----**	**----**	**18 (60.0%)**	**----**
**MWA**		**(N (%))**	**----**	**----**	**11 (36.7%)**	**----**

Chi-Square test, ANOVA*. **. P, between 3 groups. **significant (*P* value < 0.05)

Table 1 shows demographic data among the studied groups. Both controls, cirrhotic and HCC patients were matched as regard age and gender.

**Table 2:** Baseline radiological and clinical finding in HCC patients and cirrhotic patients

Parameters		Cirrhotic *N*=30	HCC *N*=50	*P*
**Number of focal lesions**	**Unifocal**	0 (0.0%)	34 (68.0%)	---
	**Multi focal**	0 (0.0%)	16 (32.0%)	
**Diameter of dominant lesion**	**Less than 5 cm**	0 (0.0%)	39 (78.0%)	---
	**More than 5 cm**	0 (0.0%)	11 (22.0%)	
**Portal vein**	**Patent**	28 (93.3%)	40 (80.0%)	0.194
	**Thrombosis**	2 (6.7%)	10 (20.0%)	
**Lymphadenopathy**	**Present**	0 (0.0%)	14 (28.0%)	**0.001**
**Extrahepatic spread**	**Present**	0 (0.0%)	9 (18.0%)	**0.023**
**Spleen**	**Average**	8 (26.7%)	20 (40.0%)	0.063
	**Splenomegaly**	15 (50.0%)	27 (54.0%)	
	**Splenectomy**	7 (23.3%)	3 (6.0%)	
**Ascites**	**Present**	5 (16.7%)	18 (36.0%)	0.064
**Ascites degree**	**Mild**	3 (60.0%)	16 (88.9%)	0.194
	**Moderate**	2 (40.0%)	2 (11.1%)	
**Child**	**A**	25 (83.3%)	39 (78.0%)	0.678
	**B**	5 (16.7%)	10 (20.0%)	
	**C**	0 (0.0%)	1 (2.0%)	
**BCLC**	**A**		26 (52.0%)	-------
	**B**		4 (8.0%)	
	**C**		15 (30.0%)	
	**D**		5 (10.0%)	

Chi-Square test (Fisher’s Exact test). P, between 2 groups. **significant (*P* value < 0.05)

Table 2 shows comparison of baseline radiological and clinical finding in HCC patients and cirrhotic patients. Portal vein thrombosis was more frequent in HCC group without statistical significance. Lymphadenopathy and extrahepatic spread were significantly frequent in HCC group. No significant result as regard child classification.

**Table 3:** Comparison of melanoma antigen genes expression among studied groups

Parameter	Control *N*=15	Cirrhotic *N*=30	HCC *N*=50	*P*	*P* ^1^	*P* ^2^	*P* ^3^
MAGE-A1	**----**	**8.89 (0.48–15.08.48.08)**	**23.19 (0.88–375.90.88.90)**	**≤0.001**	---	----	**≤0.001**
MAGE-A3	**1.02 (0.81–1.33.81.33)**	**7.10 (0.89–11.54.89.54)**	**19.26 (7.51–110.51.51.51)**	**≤0.001**	**0.027**	**≤0.001**	**≤0.001**

Continuous variables are expressed as median (min-max). Data are compared using Kruskal-Wallis. ***P***, between 3 groups; ***P***^***1***^, between control and cirrhotic; ***P***^***2***^, between control and HCC; ***P***^***3***^, between cirrhotic and HCC.**Significant (*P* value < 0.05)

MAGE-A1 expression was significantly elevated in the HCC group compared to the cirrhotic group but was below detection limit in the control group, while MAGE-A3 was significantly elevated in HCC group when compared to both cirrhotic and control groups, although it was higher in the cirrhotic group than the control group. According to the median value of each gene, HCC patients were divided into high and low gene expression groups (Table 3).

We used the median expression value within the HCC group as the cutoff to classify patients into high and low expressors. This approach avoids bias resulting from applying a diagnostic threshold (derived from ROC curve) in which the majority of HCC subjects would fall above the ROC cutoff. Using the ROC-derived cutoff would have resulted in most HCC patients being classified as “high expressors,” thereby reducing the ability to detect meaningful intra-group prognostic differences.

Both genes were elevated in patients with alpha fetoprotein (AFP) >20 ng/ml, those with tumor diameter >5 cm, subjects with multifocal lesions, cases with vascular invasion, and in patients who were classified as Child B. Conversely, these genes were expressed at lower levels when AFP was <20 ng/ml, patients had tumor diameter <5 cm or unifocal lesions, there was no vascular invasion, and in Child A patients. MAGE-A3 was significantly elevated in patients with Lymph node involvement and extrahepatic spread compared to those with no spread. With regards to BCLC classification, MAGE-A1 was significantly increased with higher stages compared to lower stages (stage A+B vs. stage D, *P*= 0.002 and stage A+B vs. stage C, *P*≤0.001), as was MAGE-A3 (stage A+B vs. stage D, *P*≤0.001and stage A+B vs. stage C, *P*≤0.001) (Table 4).

**Table 4:** Comparison of melanoma antigen genes in HCC patients as regard clinical features, tumor characteristics, and classification

Parameter		MAGE-A1 Median (Min-Max)	MAGE-A3 Median (Min-Max)	*P* ^1^	*P* ^2^
Age (years)	**<60**	16.96 (0.8–336.44.8.44)	15.97 (7.99–96.21.99.21)	0.385	0.049
	**>60**	24.32 (3.67–375.90.67.90)	24.21 (7.51–110.51.51.51)		
Gender	**Male**	28.52 (0.88–336.44.88.44)	18.87 (7.51–110.51.51.51)	0.927	0.256
	**Female**	16.96 (5.44–375.90.44.90)	24.21 (7.99–105.28.99.28)		
AFP	**<20 ng/ml**	14.62 (0.88–336.44.88.44)	14.56 (7.51–96.21.51.21)	**0.021**	**≤0.001**
	**>20 ng/ml**	39.95 (1.09–375.9.09.9)	32.18 (7.99–110.51.99.51)		
Tumor diameter	**<5 cm**	15.18 (0.8–155.87.8.87)	16.31 (7.51–110.51.51.51)	**≤0.001**	**0.001**
	**>5 cm**	104.99 (10.81–375.90.81.90)	47.77 (19.27–105.28.27.28)		
Number of focal lesions	**Unifocal**	13.49 (0.88–145.43.88.43)	15.92 (7.51–89.76.51.76)	**≤0.001**	**0.001**
	**Multifocal**	76.07 (10.59–375.90.59.90)	30.82 (11.94–110.51.94.51)		
Vascular invasion	**Absent**	14.62 (0.88–155.87.88.87)	16.54 (7.51–91.65.51.65)	**≤0.001**	**≤0.001**
	**Present**	114.78 (62.0–375.9.0.9)	78.20 (19.95–110.51.95.51)		
Lymph node involvement	**Absent**	15.55 (0.88–375.90.88.90)	16.14 (7.51–110.51.51.51)	0.056	**0.004**
	**Present**	33.69 (10.81–155.87.81.87)	41.84 (15.22–98.91.22.91)		
Extrahepatic spread (other than lymph node)	**Absent**	15.93 (0.88–375.9.88.9)	16.77 (7.51–110.51.51.51)	0.060	**0.005**
	**Present**	41.48 (10.59–105.0.59.0)	34.25 (20.09–98.91.09.91)		
Child classification	**A**	14.06 (0.88–155.87.88.87)	16.31 (7.51–98.91.51.91)	**0.002**	**0.002**
	**B**	42.21 (22.07–375.9.07.9)	48.30 (18.87–105.28.87.28)		
BCLC	**A+B**	12.62 (0.88–42.94.88.94)	14.61 (7.51–31.52.51.52)	**≤0.001**	**≤0.001**
	**C**	74.24 (10.59–228.21.59.21)	46.46 (19.95–98.91.95.91)		
	**D**	117.81 (22.07–375.90.07.90)	96.20 (28.8–110.51.8.51)		

Mann-Whitney test, Kruskal-Wallis test. ***P***^***1***^ between groups as regard MAGE-A1, ***P***^***2***^ between groups as regard MAGE-A3, **Significant (*P* value < 0.05)

**Table 5:** Comparison of melanoma antigen genes regarding treatment response in HCC patients at 24 months follow up

Parameter		CR (*N*=17)	PD (*N*=10)	*P*
MAGE-A1	**Median (Min-Max)**	**9.21 (0.88–42.94.88.94)**	**16.16 (1.09–30.79.09.79)**	**0.046**
MAGE-A3	**Median (Min-Max)**	**13.25 (7.51–24.22.51.22)**	**17.79 (11.46–31.52.46.52)**	**0.018**

Continuous variables are expressed as median (min-max). Data are compared using Mann-Whitney test. ***P***, between 2 groups. **Significant (*P* value < 0.05)

Significant elevation of MAGE-A1 and MAGE-A3 was demonstrated in progressive disease (PD) compared to complete response (CR) after different therapeutic maneuvers: trans-arterial chemoembolization, microwave ablation or percutaneous ethanol injection *(*Table 5*)*. The remaining HCC patients included 3 patients with Partial response due to small nonrepresentative group number, and 20 BCLC C+D patients were not candidate for curative modalities.

**Table 6:** Diagnostic performance of MAGE-A1 and MAGE-A3 for discrimination between HCC patients and cirrhosis patients

	MAGE-A1	MAGE-A3
**AUC**	0.781	0.966
**95% CI**	0.682–0.880	0.932–0.999
***P value***	**≤0.001**	**≤0.001**
**Cut off**	11.54	11.39
**Sensitivity (%)**	72.0%	90.0%
**Specificity (%)**	70.0%	96.7%

**Fig. 2:**
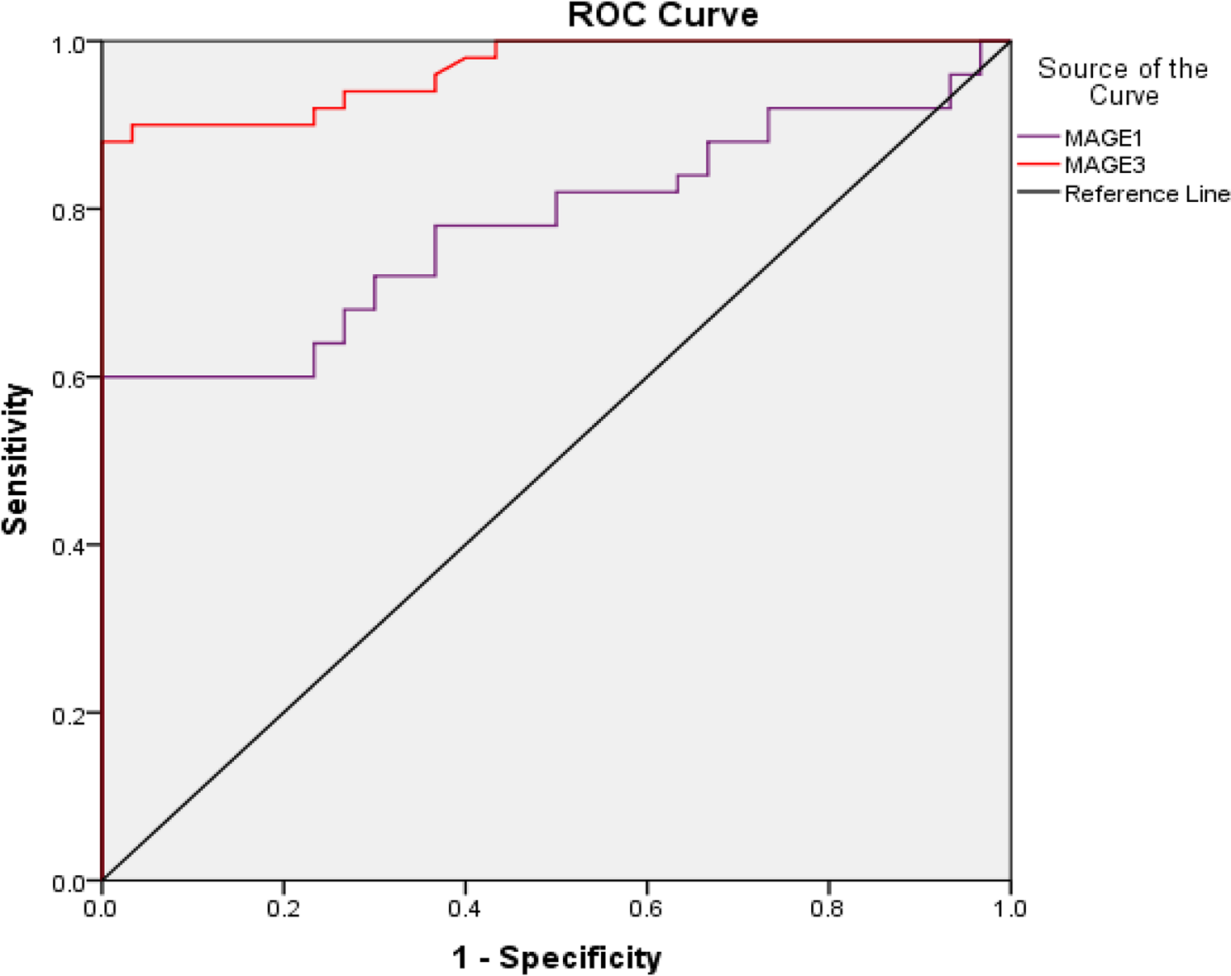
ROC curve of MAGE-A1 and MAGE-A3 for discrimination of HCC from cirrhosis

ROC analysis was conducted for both genes in discrimination between HCC and cirrhosis patients, showing that the best cut-off value for MAGE-A1 was above 11.54 in predicting HCC from cirrhosis, with area under the curve (AUC) of 0.781 (*P*≤0.001). Similarly, the best cut-off value for differentiating HCC from cirrhosis by MAGE-A3 was above 11.39, with AUC of 0.966 (*P*≤0.001) (Table 6; Figure 2).

**Table 7:** Cox regression analysis to predict hazardous factors that affect overall survival (OS) in HCC patients

Parameter	Univariable			Multivariable		
	*P*	HR	95% CI	*P* value	HR	95% CI
**Age**	0.718	1.013	0.944–1.088			
**Gender**	0.266	0.485	0.135–1.739			
**Tumor diameter ** **(>5 cm vs. <5 cm)**	**≤0.001**	**7.453**	**2.464–22.547**	0.758	1.249	0.305–5.115
**PV invasion**	**≤0.001**	**20.411**	**5.240–79.503**	0.064	4.413	0.917–21.233
**Lymph node involvement**	0.332	1.728	0.573–5.213			
**Extrahepatic spread (other than lymph node)**	0.461	1.650	0.436–6.244			
**AFP**	**0.048**	**3.291**	**1.010–10.725**	0.814	1.853	0.626–3.210
**MAGE-A1**	**0.003**	**23.096**	**2.962–80.096**	**0.048**	**9.407**	**1.023–86.526**
**MAGE-A3**	**0.003**	**22.063**	**2.810–73.234**	**0.042**	**9.199**	**1.894–94.694**
**AFP +MAGE-A1**						
**•Both low (16)**: Reference						
** •High MAGE-A1 and low AFP (8)**	**0.032 **	**7.182**	2.574-28.143	0.134	1.157	0.697-14.498
**•Low MAGE-A1 and high AFP (9)**	0.941	1.212	0.967-3.508			
**•Both high (17)**	**0.029**	**10.468**	1.493-23.589	0.188	3.104	0.707-13.464
**AFP+MAGE-A3**						
**•Both low (19)**: Reference						
**•High MAGE-A3 and low AFP (5)**	**0.018 **	**15.356 **	1.595-47.820	0.067	9.296	0.854-31.229
**•Low MAGE-A3 and high AFP (5)**	0.986	1.986	0.991-3.578			
**•Both high (21)**	**0.007**	**18.262**	** 2.228-49.747**	** 0.039**	**10.681**	1.132-50.818

*HR* Hazard ratio, *CI* Confidence interval, (r): Reference group

AFP is considered high or low at levels above or below 20 ng/ml

MAGE-A1 expression is considered high or low at levels above or below 23.19

MAGE-A3 expression is considered high or low at levels above or below 19.26

COX regression analysis conducted to predict factor(s) affecting OS using age, gender, tumor diameter, PV invasion, LN involvement, extrahepatic metastasis, AFP, and MAGE genes as covariates. Univariate analysis demonstrated significant independent factors for shorter OS in HCC patients to be tumor diameter >5 cm, PV invasion, increased AFP, elevated MAGE-A1, and high MAGE-A3. In addition, high levels of MAGE-A1, high MAGE-A3, and increased AFP and MAGE-A3 levels were independent factors for shorter OS in HCC patients using multivariate analysis (Table 7).

The survival analysis was performed on the entire HCC cohort (*n* = 50), not only the treatment subgroup. All 50 patients were followed up for 24 months, and their OS outcomes were available for the Cox regression analysis. Therefore, the event number was sufficient to allow a stable multivariate model.

## Discussion

Despite recent advance in HCC diagnosis and therapeutic modalities, the mortality rate of HCC remains surprisingly high; this may be partially attributed to the consistent delays in HCC diagnosis hindering the efficacy of the available curative modalities [22]. As a result, with the aim of enhancing survival and improving patient quality of life, this has highlighted the need for novel diagnostic and prognostic biomarkers.

To assess expression levels of MAGE-A1 and MAGE-A3 genes in HCC and cirrhosis, the current study demonstrated significantly higher levels in patients with HCC in comparison to liver cirrhosis and to healthy volunteers who showed no expression of MAGE-A1 gene but very low MAGE-A3 expression levels. Similar findings were reported by one of the earliest groups to investigate MAGE family gene expression in HCC patients, but unlike blood samples taken in the present study, the original study took samples from tumor and adjacent non-cancerous liver tissue [23]. Comparable results were subsequently confirmed in peripheral blood in later studies, suggesting the potential role of both genes as novel markers for early HCC detection [24].

Epigenetic regulation through promoter hypomethylation and histone modification by manipulating lysine residue appears to play a major role in releasing the silenced state of MAGE genes and re-expression in tumor cells [25].

Several mechanisms have been elucidated as regards the role of MAGE genes in cancer evolution; MAGE proteins form complexes with E3 ubiquitin ligases which in turn alter the activity of the tumor suppressor AMPKα1 (5’adenosine mono-phosphate activated protein kinase) leading to loss of autophagy [26], beside induction of P53 degradation and mammalian target of rapamycin (mTOR) hyperactivation resulting in loss of growth control and enhancing tumor initiation and progression [27].

Noteworthy is the detection of minimal MAGE-A3 gene expression in healthy controls in the current study, a finding contradictory to previous reports stating that MAGE A subfamily members, including MAGE-A3, were not expressed in normal subjects with expression being restricted to patients with malignancies [28, 29]. However, some studies have reported low-level expression of different MAGE I subfamily members in non-malignant patients and MAGE-A3 was additionally expressed in two patients with HCV and liver cirrhosis in a study evaluating MAGE gene mRNA expression [24].

Expression patterns of MAGE-A1 and MAGE-A3 genes were found to be capable of distinguishing HCC from liver cirrhosis, in addition to corresponding to HCC phenotype, with MAGE-A1 expression related to tumor size, number of nodules, vascular invasion, alpha fetoprotein secretion, and tumor stage according to BCLC staging system, while MAGE-A3 expression was additionally associated with extrahepatic metastatic spread and Lymph node involvement. These findings coincide with results by Teama et al., 2013 [30] reporting higher levels of MAGE-A1 and MAGE-A3 mRNA expression in patients with metastatic HCC compared to primary HCC patients; hence the current study may represent further validation of such results with relatively larger sample size (50 versus 46 HCC patients, 30 versus 12 cirrhotic patients and 15 healthy volunteers in both studies). Points of contrast between the two studies included classifying cirrhotic patients into 25 child A and 5 Child B patients and classifying HCC patients according to BCLC staging system versus no cirrhotic group subclassification and using the relatively vintage TNM system in the corresponding study.

Additionally, the study of Teama et al., 2013 relied on patients with relatively larger tumor burden (21 of 32 HCC patients had lesions >5 cm) versus 39 HCC patients in our study had dominant lesions <5 cm confirming the valuable use of both genes in HCC diagnosis at earlier stages. Finally and the most promising point is linking expression levels of both genes to response to therapy and OS in HCC patients. Consequently, expression patterns of these two genes may be used as markers of circulating tumor cells to predict early tumor vascular invasion or extrahepatic spread.

In addition, a previous study evaluating expression patterns of the two genes in HCC patients revealed a strong prognostic value for both, as evidenced by increased mortality due to recurrence and/or metastatic disease in patients manifesting persistent gene expression following curative resection or in patients positive for expression after being initially negative when compared to patients with negative gene expression [31].

In cancer cells, MAGE-A family members become dysregulated and acquire tumor promoting characteristics, including enhanced cellular proliferation, tissue invasion, and migration, concurrently with inhibited apoptosis [32].

A novel addition of this study was linking the expression levels of both MAGE genes to the response to therapeutic intervention, revealing significantly higher expression in patients with progressive disease than in patients with complete response, thus confirming the role of these genes as markers of prognosis in HCC [28, 31].

In order to identify hazardous factors that may influence OS of HCC patients, COX regression analysis was performed showing that MAGE-A1 and MAGE-A3 genes were significant independent predictors of shorter OS. Poojary et al., 2020 [33] shared similar findings, thus confirming the role of these genes in HCC, and providing insight for their application as gene therapy [34, 35]. Other independent factors predicting shorter OS in HCC were found to be tumor size larger than 5 cm, portal vein invasion, and high alpha fetoprotein levels. Furthermore, multivariate analysis showed that high expression of MAGE-A1, increased MAGE-A3, and elevation of both AFP and MAGE-A3 gene were identified as independent covariates predicting shorter OS in HCC.

To evaluate the adequacy of the sample size for the treatment response subgroup, a post-hoc power analysis was performed using the observed data for MAGE1 and MAGE3 expression between complete-response (CR, *n* = 17) and progressive-disease (PD, *n* = 10) patients. The analysis indicated that the study had approximately 78% power (α = 0.05) to detect the observed difference in MAGE3 expression (Cohen’s d = 1.13), representing a large effect size and confirming adequate statistical sensitivity for this comparison. In contrast, the power for MAGE1 was only 5% (Cohen’s d = 0.05), reflecting a negligible effect and high variability between individuals. These findings suggest that the significant association observed for MAGE3, but not for MAGE1, is supported by sufficient statistical power. The findings should be validated in larger, multicenter cohorts.

This study concluded that MAGE-A1 and A3 genes can be used as markers for early HCC diagnosis and prognosis, especially in patients with advanced tumor stage or progressive disease. Higher levels of these genes either alone or with high AFP can predict shorter OS in HCC patients.

For the preceding facts, MAGE-A1 and A3 genes have gained a reputation as promising markers for early HCC detection and discrimination from liver cirrhosis. In addition to risk stratification and predicting therapeutic response and patient survival in HCC.

Among the limitations of the present study was that it was performed at a single center on a relatively small group of patients specifically after HCC group subdivision based on treatment response, lacking external validation, limited ethnic diversity of study population, potential selection bias and lack of comparison with other biomarkers. Moreover, the common risk factor for HCC development in this study was HCV-induced liver cirrhosis, not taking into consideration other causes of HCC, such as HBV or non-alcoholic fatty liver disease (NAFLD), which could possibly influence MAGE gene expression.

## Supplementary Information

Supplementary Material 1

Supplementary Material 2

Supplementary Material 3

Supplementary Material 4

## Data Availability

The datasets used and/or analysed during the current study are available from the corresponding author on reasonable request and are available online on Ibn-Sina online data system of Mansoura University Hospitals website: [https://srv137.mans.edu.eg].Patient data supporting the findings of this study are available as supplementary file with this manuscript. All data have been fully de-identified to protect patient privacy in compliance with ethical standards and applicable data protection regulations.

## Abbreviations

AFP: Alpha fetoprotein
AMPKα1: 5’adenosine mono-phosphate activated protein kinase
AUC: Area under the curve
BCLC: Barcelona-Clinic Liver Cancer
cDNA: Complementary deoxyribonucleic acid
CR: Complete response
CSC: Cancer stem cell
DNA: Deoxyribonucleic acid
EASL: European Association for the Study of the LIVER
GAPDH: Glyceraldehyde-3-phosphate dehydrogenase
HBV: Hepatitis B virus
HCV: Hepatitis C virus
HCC: Hepatocellular carcinoma
IRB: Institutional Research Board
MAGE: Melanoma Antigen Gene
mRECIST: Modified Response Evaluation Criteria in Solid Tumors
mTOR: Mammalian target of rapamycin
NAFLD: Non-alcoholic fatty liver disease
OS: Overall survival
PBMCs: Peripheral blood mononuclear cells
PD: Progressive disease
qRT-PCR: Quantitative real-time polymerase chain reaction
RNA: Ribonucleic acid
RQ: Relative Quantity
