# Supplementary material for: Melanoma antigen genes A1 and A3 as predictors of treatment response and survival in HCV-associated hepatocellular carcinoma: a prospective study
Source: BMC Gastroenterol. 2026 Jan 22;26:68. doi: 10.1186/s12876-025-04574-8 (PMC12836994; doi:10.1186/s12876-025-04574-8)
Supplement: Supplementary file 2 — Supplementary Material 2 [file 12876_2025_4574_MOESM2_ESM.docx]

**STROBE Statement for the Manuscript providing a structured summary of what should be included in each section of the manuscript to ensure transparent and complete reporting**

Title and Abstract:

· 1 (a) Title: Indicate the study’s design with a commonly used term (e.g., "prospective cohort study").

· 1 (b) Abstract: Provide a balanced summary of what was done and what was found including the rationale for investigating MAGE-A1/A3 in HCV-HCC. The objective to assess the association between MAGE-A1/A3 expression and treatment response and overall survival, main results of the study and finally a conclusion of main findings.

Introduction:

· 2 Background/rationale: about the burden of HCV-associated HCC and MAGE-A family members.

· Explain the need for MAGE-A1 and A3 as predictive biomarkers for treatment response and survival in HCC

· 3 Objectives: Specific objectives and hypotheses.

· State the primary objective: To prospectively evaluate the association between pre-treatment MAGE-A1/A3 expression and response to first-line therapy and overall survival.

Methods

· 4 Study design: Present key elements of study design early in the paper "prospective case-control study."

· 5 Setting: Describe the setting, locations, and relevant dates, including periods of recruitment, exposure, follow-up, clinical centers involved (Mansoura university tropical medicine department in collaboration with medical biochemistry and radiology department, from 2020 to 2024, 2 year follow up duration).

· 6 Participants:

· (a) Eligibility Criteria were clearly defined (e.g., adult patients with HCV-associated HCC, eligible for standard therapy (MWA, TACE, PEI, systemic therapy).

· (b) Sources and methods of participant selection: participants are selected randomly after sample size calculation.

· 7 Variables: Clearly define all outcomes, exposures, predictors, potential confounders, and effect modifiers.

· Exposure: MAGE-A1 and A3 expression measured by quantitative RT-PCR with the cut-off point for "high" vs. "low" expression.

· Outcomes: Treatment Response defined by mRECIST criteria assessed by blinded radiologists at a predefined time point (e.g., 3 months after treatment initiation) and overall Survival (OS): Time from diagnosis/treatment initiation to death from any cause.

· Confounders: Identify key confounders for which statistical adjustment was made. These likely include: age, sex, Child-Pugh grade, BCLC stage, tumor size/number, alpha-fetoprotein (AFP) level, and type of first-line treatment received.

· Effect Modifiers: Consider if any variables (e.g., BCLC stage, treatment type) might modify the effect of MAGE-A on outcomes and plan stratified analyses.

· 8 Data sources/ measurement: For each variable, give details of methods of assessment and sources of data.

· Clinical data (staging, liver function, treatment) were collected (e.g., prospective electronic database, patient charts).

· Detail the laboratory methods for MAGE-detection (PCR primers, assay conditions).

· 9 Bias: the prospective design to minimize recall and selection bias, blinding of pathologists and radiologists.

· 10 Study size: Sample size was calculated using online sample size calculator (https://riskcalc.org/samplesize/) with anticipated prevalence of MAGE1 and MAGE3 gene expression in HCC as regard cirrhosis and healthy volunteers

· 11 Quantitative variables:

· We used the median expression value within the HCC group as the cutoff to classify patients into high and low expressors. This approach avoids bias resulting from applying a diagnostic threshold (derived from ROC curve)

· 12 Statistical methods:

· Data were entered and analyzed using IBM-SPSS software (IBM Corp. Released 2017. IBM SPSS Statistics for Windows, Version 25.0. Armonk, NY: IBM Corp.).

Bonferroni correction was applied to control for multiple testing; however, because several comparisons yielded very small p-values (p < 0.001), the corrected results remained significant.

Stepwise Cox regression confirmed MAGE1, MAGE3, and AFP as the most stable independent predictors of shorter OS.

Results

· 13 Participants:

· (a) Report numbers of individuals at each stage of the study: out of 150 HCC patiients50 were included to assess MAGE gene expression.

· (b) Give reasons for non-participation at each stage.

· (c) A Flowchart of included patients is added.

· 14 Descriptive data:

· (a) Characteristics of study participants (e.g., demographic, clinical, radiological) included as baseline table.

· (b) Indicate the number of participants with missing data for each variable of interest.

· 15 Outcome data:

· Report numbers of outcome events for each primary outcome, for survival analysis, report the number of events, the median follow-up time (2 years), and survival probabilities at key time points (e.g., 1-year, 2-year OS).

· 16 Main results:

· Reported unadjusted and adjusted estimates (e.g., OR, HR) with confidence intervals. Making clear which confounders were adjusted for and why.

· 17 Other analyses: Report results of any other analyses performed.

Discussion

· 18 Key results were summarized with reference to study objectives.

· 19 Limitations of the study were discussed, taking into account sources of potential bias.

· 20 Interpretation: A cautious overall interpretation of results was given considering objectives, limitations, multiplicity of analyses, results from similar studies, and other relevant evidence

· 21 Generalisability: lack of external validitation and generalisability of the study findings.

· 22 Funding: The source of funding was given with the role of the funders in the present study.

· Ethical Approval: State that the study was approved by an institutional review board and that all participants provided informed consent.
