# Supplementary material for: Melanoma antigen genes A1 and A3 as predictors of treatment response and survival in HCV-associated hepatocellular carcinoma: a prospective study
Source: BMC Gastroenterol. 2026 Jan 22;26:68. doi: 10.1186/s12876-025-04574-8 (PMC12836994; doi:10.1186/s12876-025-04574-8)
Supplement: Supplementary file 3 — Supplementary Material 3 [file 12876_2025_4574_MOESM3_ESM.pdf]

Consent form

I …………………………………………………………………………………... [Name] give my consent for information about myself/my child or ward/my relative (circle as appropriate) to be published in BMC Gastroenterology, Manuscript ID: 2419dca1-e0f7-41ad-9e1c-e0b4ba29b375, corresponding author: Amr Samir.

I understand that the information will be published without my/my child or ward's/my relative's (circle as appropriate) name attached, but that full anonymity cannot be guaranteed.

I understand that the text and any pictures or videos published in the article will be freely available on the internet and may be seen by the general public. The pictures, videos and text may also appear on other websites or in print, may be translated into other languages or used for commercial purposes.

I have been offered the opportunity to read the manuscript.

Signing this consent form does not remove my rights to privacy.

Name…………………………………

Date………………………………….

Signed………………………………...

Author name: Amr Samir

Date: 28-7-2025

Signed Amr Samir

Please keep this consent form in the patient's case files. The manuscript reporting this patient's details should state that 'Written informed consent for publication of their clinical details and/or clinical images was obtained from the patient/parent/guardian/ relative of the patient. A copy of the consent form is available for review by the Editor of this journal.

BioMed Central Limited, 236 Gray's Inn Road, London WC1X 8HL, UK
BioMed Central Limited is part of Springer Science+Business Media. VAT No. GB 823 8263 26 Registered in England and Wales No. 3680030

T +44 (0) 20 3192 2000 **F** +44 (0) 20 3192 2010 **W** www.biomedcentral.com **E** info@biomedcentral.com
