# Supplementary figures and images for: Melanoma antigen genes A1 and A3 as predictors of treatment response and survival in HCV-associated hepatocellular carcinoma: a prospective study
Source: BMC Gastroenterol. 2026 Jan 22;26:68. doi: 10.1186/s12876-025-04574-8 (PMC12836994; doi:10.1186/s12876-025-04574-8)

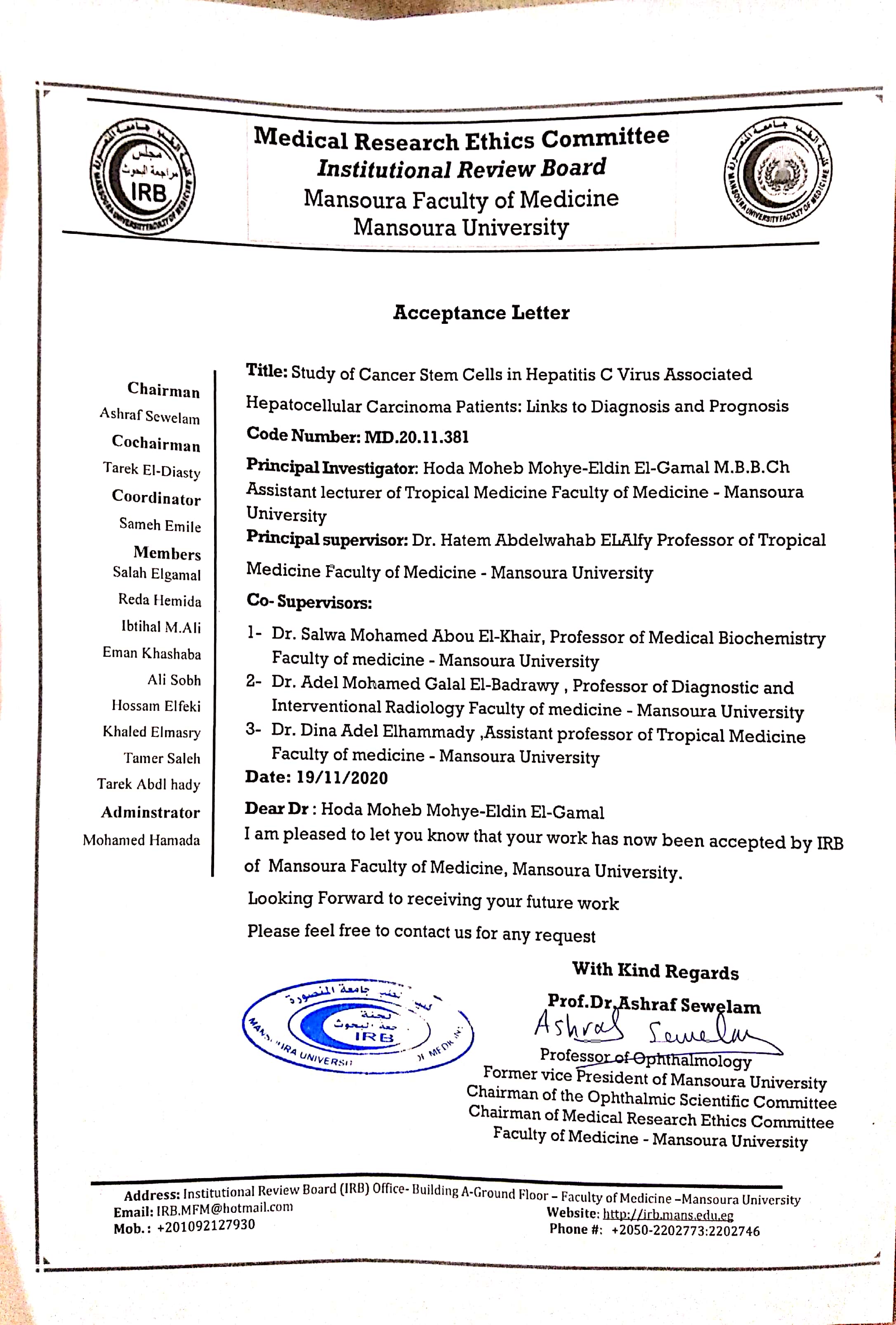

Supplement: Supplementary file 4 — Supplementary Material 4 [file 12876_2025_4574_MOESM4_ESM.jpg]
